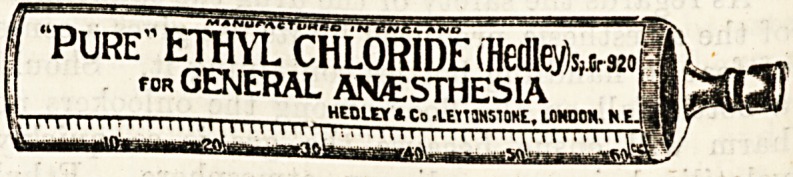# Ethyl Chloride Anæsthesia: Practical Points in Its Administration

**Published:** 1906-10-27

**Authors:** W. Lauzun-Brown

**Affiliations:** Anæsthetist to the Central London Hospital for the Throat, Nose, and Ear; formerly Civil Surgeon, Netley Hospital; Medical Officer, Ashanti Field Force


### NEW ANÆSTHETIC METHODS.

### ETHYL CHLORIDE ANÆSTHESIA: PRACTICAL POINTS IN ITS ADMINISTRATION.

By W. Lauzun-Brown, L.R.C.P., L.R.C.S. (Edin.), Anæsthetist to the Central London Hospital for the Throat, Nose, and Ear; formerly Civil Surgeon, Netley Hospital; Medical Officer, Ashanti Field Force.

(*Continued from page* 54.)

In addition to the inhalers already mentioned that by Mr. Vernon Knowles is of exceptional interest. It is a little complicated, but can readily be taken to pieces and has valves for the regulation of the admission of air controlled by a lever. The anæsthetic can be admitted gradually and this tends to obviate struggling and holding the breath, so embarrassing to the administrator. The narcosis is lengthened by the admission of air and the after-effects are minimised. There is also an attachment by which capsules with a measured quantity may be employed—a very important matter in administering the anæsthetic in warm climates. The apparatus has the wide bore and may be fitted to Hewitt's ether apparatus, where the ethyl chloride and ether sequence is desired.

### History as a General Anæsthetic.

The administration of ethyl chloride has an interesting history. It was first employed by Heyfelder in 1848, and in this country by Sir Benjamin Ward Richardson, who employed it as a general anæsthetic in 1867, and declared it to be "a good safe anæsthetic." His report attracted no attention among the anæsthetists of that time. Twenty years later English dentists began to use it, but an adverse report by a Committee of the British Medical Association delayed its employment in this country. In 1895 Carlson froze teeth with it at Dental Institute of Gothenburg. He noticed that it frequently rendered the patients unconscious. This induced some persons to use it as a general anæsthetic with excellent results. In Lyons, where it is extensively manufactured, numberless administrations were reported. In 1897 and 1898 it was systematically used in Von Hacker's clinic. And so on the progress went until ethyl chloride found its way once more into England and was used as a general anæsthetic by McCardie, of Birmingham, who in 1903 published a paper on a collection of cases.

Ethyl chloride has many qualities to recommeid it. It is portable, stable, easy to administer, not bulky, requires no very complicated apparatus and produces a rapid, quiet narcosis lasting for several minutes, terminating in a rapid recovery, with few, if any, unpleasant or dangerous symptoms.

### Some of its Properties.

As regards the safety of the drug the suddenness of the anæsthesia produced by its use gives a sense of fear in handling bottles containing it. Should a bottle fall on the floor among the onlookers no harm will ensue because the gas is so quickly volatilised in an ordinary atmosphere. Ethyl chloride sprayed into the fire burns with a greenish flame on account of the chlorine. If a bottle should by any chance be broken and the vapour catch fire it may be extinguished by the doctor's coat. It will not burn the garment, nor the carpet, or any dress on which it may happen to fall. When disinfecting the face-piece remove it from the inhaler. All metal tubing connected with its administration should be carefully cleaned. A point of interest to the country practitioner is not to carry the bottle in the vest pocket, for if it breaks there is some risk of catching cold from the sudden cooling of the surrounding parts. When swallowed it is a strong intoxicant.

### How to Recognise an Absolute Ethyl Chloride.

Ethyl chloride is prepared by the action of hydrochloric acid on ethyl alcohol. Pure ethyl chloride can be distinguished by its agreeable ethereal odour. If a little of the ethyl chloride be allowed to evaporate from the hand, no disagreeable smell should be perceptible while the liquid is evaporating. A little practice in this test with a pure and impure sample readily makes one proficient in detecting the odorous impurities. Ethyl chloride should not have an acid reaction to litmus. The acid most generally present, from faulty manufacture or purification, is hydrochloric, but, under some circumstances, sulphurous acid may also be present. The ethyl chloride should not redden blue litmus paper if allowed to lie in contact with it for at least fifteen minutes. Hydrochloric acid may also be detected by shaking the ethyl chloride in a separator with half its volume of water. On separating the water and adding a few drops of silver nitrate solution, no precipitate or opalescence should be produced. It boils at 12.5° C., and has a specific gravity of 0.921 at 0° C.

A gravity lower than this might be due to the presence of ether. The latter has not necessarily been added, but indicates carelessness in the method of manufacture. In this respect it may be pointed out that an anæsthetic ethyl chloride of French manufacture is stated in a circular to have a specific gravity of 0.874 at 5° C., with a boiling point at 11° C. The following is the method of detecting foreign organic compounds decomposable by sulphuric acid: If the ethyl chloride be shaken in a separator with a fifth of its volume of pure colourless concentrated sulphuric acid, the acid on separation should be colourless. If the acid be yellow coloured it indicates the presence of empyreumatic bodies, foreign to the ethyl chloride, which is unacted upon by sulphuric acid, unless on very long standing. Acid thus coloured by an impure ethyl chloride often evolves an unpleasant odour on addition of five times its volume of water. Ether, if present, is also readily detected by the odour. The different specific gravities as recorded by different observers at different temperatures are: Specific gravity at 0 per cent. C., 0.9214; specific gravity at 2 per cent. C., 0.9229; specific gravity at 6 per cent. C., 0.917; specific gravity at 8 per cent. C., 0.9176. Above the boiling point (12.5 per cent. C.) it is of course a gas. Ethyl chloride is very slightly soluble in water, but dissolves readily in alcohol. It is inflammable when brought in contact with a light, and is decomposed, burning with a green-edged flame. It should not be administered near a light. As regards the action of air and light, it is probable that ethyl chloride is in the same category as chloroform—that is to say, it can be kept unchanged if exposed to air and light singly, but the combined action affects its purity.

### Its Range of Usefulness.

This anæsthetic is eminently suited for dental operations requiring an anæsthesia of a minute and a half or two minutes. Frequently as many as eight or nine teeth can be extracted with one administration. At the Central London Throat and Ear Hospital it is extensively used for out-patient operations, such as removal of tonsils, adenoids, nasal polypi, turbinate bones, and so forth. One operator last week removed at one sitting with one administration of ethyl chloride in the same patient the lower turbinate, both tonsils, and a considerable pad of adenoids, stopping towards the end of the operation to clear the Eustachian tubes. This required time to do, and yet the anæsthesia was perfectly sufficient for the purposes of the operator, and the recovery from the anæsthetic in no way complicated.

Besides having a wide range in dentistry, it plays a considerable role in minor surgery. Small tumours may be removed, a whitlow lanced, a bubo incised, a hydrocele injected, a stricture stretched, an ulcer cauterised, the abdomen or chest aspirated, and fractures or dislocations may be reduced under ethyl chloride anæsthesia. Painful examinations may be made, urethral caruncles destroyed, vulvo-vaginal abscesses opened, vaginal cysts extirpated, the uterine cervix dilated, the uterus curetted, and mammary abscesses may be opened and drained. The sphincters may be dilated, fissures incised, hemorrhoids ligated, fistulæ laid open and perirectal abscesses drained. The ophthalmologist will be able to suggest many cases in which it may be found useful. In nose and throat practice it acts splendidly in the removal of tonsils and adenoids and operations on the nasal cavities.

### Preliminary Matters.

A properly constructed face-piece is an essential to its correct administration. The face-piece best adapted for ethyl chloride is that supplied by Messrs. Duncan Flockhart and Company. It is a transparent celluloid face-piece enabling the administrator with perfect ease to watch the breathing, the lips, and the tongue. The air-cushion around it is prevented from slipping off by being attached by a series of buttons. Nothing is more disconcerting to the administrator than to find the celluloid part of the face-piece slipping past the air cushion on to the patient's face, and permitting the entrance of air during the administration when it should be excluded. As the administration proceeds saliva is secreted and acts as a lubricant between the celluloid and the air-cushion, and causes the india-rubber part to slip. The use of this face-piece gives the administrator a sense of security. Maw's face-piece is constructed on an identical principle. Nearly all modern face-pieces are of celluloid. Other kinds cannot be recommended. In no circumstances should ethyl chloride be given without a proper gag being first carefully adjusted. The gag should lie close to the cheek without pressing on it or injuring it in any way, and to prevent the entrance of air when the face-piece is applied should rest on a pad of cotton wool. There is no need to dilate the gag inconveniently at the outset, for when muscular relaxation occurs the mouth can be opened wider and the gag fixed so as to enable the operator to proceed. Doyen's gag is the most suitable although it is not perfection in every respect. Modifications of it have been devised by Dr. George and by Mr. Probyn Williams. In Probyn Williams' gag a metal tube is fixed to the lower blade through which chloroform may be administered. This arrangement is not quite suitable for a continuous administration of ethyl chloride. A modification of this gag has been made for me by Messrs. Duncan Flockhart and Company, in which the delivery tube as suggested by Dr. Jakins has been placed on the upper blade of the Doyen, and there opens out into a flat perforated box-like structure to prevent the ethyl chloride passing into the mouth in a liquid form.

### Ethyl Chloride Tubes.

Ethyl chloride is difficult to manage on account of its volatility, its low boiling-point, and the suddenness with which it vaporises. At first the containers were, and some still are, made of metal; but it was not found convenient in every respect, and glass containers were used. The difficulty about glass bottles was to find a suitable stopper. Hitherto none have been found so good as that supplied by Duncan, Flockhart and Company. The construction of their stop-cock is so perfect that leakage is avoided, plugging of aperture is obviated, and a steady continuous stream of the drug, either in coarse or fine spray, is ensured. The tubes are graduated in cubic centimetres, of which each tube contains sixty.

The firm of Hedley and Company, Leytonstone, has also introduced a very convenient bottle which should serve every purpose. There are many other ethyl chloride bottles on the market, but none of them seem to have any advantages over the two mentioned.

### The Preparation of the Patient.

The preparation of the patient is much the same as for chloroform or ether. The presence of food in the stomach causes vomiting, and this is greatly increased by blood passing into that organ. The bladder should be thoroughly emptied just before the patient is brought into the operating-room, as involuntary defæcation and micturition occur during, or immediately after, the narcosis. The clothing about the neck should be loosened, all false teeth removed and all surgical preparations for the operation should be complete before the administration of the anæsthetic begins. It is best to teach the patient to breath nicely, regularly and quietly before commencing the administration. In this respect Hedley and Company's inhaler has immense advantages. The face-piece can be closely applied, and the ethyl chloride sprayed into the drum, and yet the patient breathes nothing but fresh air. By shifting the lever forwards a mixture of air and ethyl chloride can be obtained and kept up until the patient becomes accustomed to the vapour; when the lever is pulled forward pure ethyl chloride only is inhaled, while all communication of the surrounding atmosphere is shut off and the patient exhales into the bag. This is a very great advantage.

The patient, previously prepared, is seated in the operating-chair. To prevent him falling forward during narcosis a leather strap is drawn across the thighs and another across the legs to prevent kicking. As the masseter muscles are apt to become rigid a Doyen's gag should be carefully applied before the administration, special care being taken not to injure the patient's lips or teeth. The face-piece is applied gently and carefully, and the patient asked to breathe quietly in the ordinary manner. The ethyl chloride is sprayed into the receptacle—in Dr. George's inhaler the sphere, in Hedley's the drum, in Dr. Kingsford's the bag, and in Mr. Luke's (Duncan Flockhart and Company's) the bag. The anæsthetist should see that the face-piece does not admit air. In from thirty-three to forty or fifty seconds the anæsthesia should be complete and the face-piece removed for the operation to proceed.

### What are the Tests for Complete Anæsthesia?

Graduates at the Central London Throat and Ear Hospital often ask how to tell when anæsthesia is complete. The experienced administrator knows by practice from a combination of signs and symptoms when the patient is ready. The first indication is a slight quivering of the eyelid. There ought to be no struggling in a properly graduated administration. The stage of quiet breathing, gradually deepens until stertor is reached. If the eyeball is examined it will be found that the pupil may or may not react to light, that later the pupil becomes dilated and fixed, and that the corneal reflex is abolished. Complete muscular relaxation indicates that the anæsthesia is complete. In screaming children, too much of the vapour should not be permitted to be inhaled at once, otherwise the anæsthesia may be too sudden and too profound, and produce effects which will surprise and startle the tyro or novice. The inhaler should be removed for a second or two to permit the excess vapour to volatilise, which it very quickly does. The breathing then resumes its natural course and the anæsthesia proceeds normally. Another difficult patient is the excitable neurotic. These persons get into a state of terror and try to struggle. This can be avoided by tact, promptness in applying the gag, and in placing the bag over the patient's face and getting a good supply quickly into the lungs. The wide-bore appliance of Dr. Kingsford, or that of Wyley and Co., of Coventry, is most suitable for these cases, and also for alcoholics. Care should be taken to see that the hands of the patients are secured, or the inhaler will be snatched off before the administrator can prevent it.

### The Question of Dosage.

It is impossible for an anæsthetist to say what dose of chloroform or of sulphuric ether is required to produce narcosis in any given case. It is exactly similar with ethyl chloride. The phenomena are produced so rapidly that it is as well to indicate, for the safety of the practitioner, the approximate quantities that are needed in various cases. This depends greatly on the inhaler used, some instruments being more wasteful of the anæsthetic than others, those in which it is sprayed into the bag being the worst offenders in this respect. For infants and young children about 2 cc., up to ten years 3 cc., and above that from 4 to 5 cc. Ethyl-chloride bottles are provided with a graduated index. The practitioner should learn how many seconds it takes to spray these various quantities from the bottle into the inhaler. *The great point is to watch the effect and to remove the inhaler whenever anæsthesia is as complete as is required for the operation.* The administrator, too, should never be in too great a hurry, and should carefully watch the breathing. If it stops at the beginning of the administration remove the inhaler and loosen the gag, and wait until it starts again before replacing the face-piece. Safety lies in unremittingly watching the condition of the patient. The dose varies in various individuals, thus a big robust man requires a larger dose than a small delicate woman. Alcoholics and excessive smokers and athletes require more than the normal dose, while sickly and anæmic patients require less. Hysterical patients usually require a large dose.

The Dangers.

Every anæsthetic has its special danger, and ethyl chloride is no exception to this rule. Its chief danger consists in clumsy, unskilful, careless administration arising out of a want of familiarity with the powers of the anæsthetic. All sorts of terrors have been conjured up in the name of ethyl chloride to frighten this excellent anæsthetic agent out of existence. Almost as many and as serious accusations have been alleged against it as were made against chloroform in the early days of its application. To us nowadays the objections then raised appear exceedingly foolish, but at the time they had a real influence in delaying the use of anæsthetics. Every anæsthetic has to pass through the stage of criticism, and ethyl chloride can form no exception to this rule. There need be no unusual dread about its administration. Criticisms which have been urged against it readily break down, and a little practice and familiarity with the tricks of the new agent usually make converts of critics. There are real dangers, however, and it is for that reason that one should strenuously insist that a knowledge of the drug should be obtained before its administration is attempted. One is almost inclined to add that in case of accident the fault is due to the administration, or to the patient, rather than to the anæsthetic. One critic, obviously unfamiliar with it, notices as one of its evil effects rigidity coming on after the anæsthesia is over, and alludes to the patient's inability to unlock the fingers as a "spasm," "great force being necessary to relax the same." The usual practice in the Central London Throat Hospital is to make the patient interlock the fingers before the administration. They naturally remain in that condition during the process of recovery, and thus tend to prevent spasmodic and jerky movement of the limbs. The after-rigidity of a patient who had obviously not been properly secured in the operating-chair has been alluded to as another spasm. All the bad effects could have been avoided by a careful watching of the respiration, and by removing the face-piece for a second or two during the anæsthesia. These cases were common enough in the early days, and seem to be induced by a too sudden and voluminous administration of the vapour and an unnecessary admission of air at the wrong time. In this hospital where the ethyl chloride is given some scores of times every week, conditions of that kind are very seldom met with. One critic goes the length of saying that ethyl chloride is exceedingly dangerous to administer in operations affecting the nasal passages. More than 7,000, to take a very rough estimate, administrations have been given at the Central London Throat and Nose Hospital for operations of these very passages, and no indication of the kind has ever been noticed. Some maintain that in older patients it is better to give ether and chloroform in certain nose cases, but this is entirely negatived by the practice at the Central London Throat Hospital. Of course there are many surgeons who desire a longer anæsthesia in these cases, and who prefer gas and ether. That is always at hand should it be desired.

As an instance how easy it is to attribute fatalities to a particular anæsthetic, I may relate the following case which occurred last week. A child sixteen months old the subject of adenoids was brought into the out-patients' operating-room of the Central London Hospital, and, being seated on a nurse's knee, the gag was applied and about four drops of ethyl chloride were placed into the inhaler. The face-piece was held for about fifteen seconds in front of the child's mouth, as it was expected the operation would be extremely brief. As soon as the effects of the anæsthetic began to be felt, the palate became very relaxed, leaving hardly any room for breathing. During the operation the patient became blue and ceased to breathe. This condition was promptly noticed, the child was inverted and the gag replaced, the tongue was pulled forward and on examining the pharynx with the fore-finger the tissues were found swollen and relaxed, completely preventing the child from breathing. Cold affusions, artificial respiration, and dilatation of the respiratory apertures by means of dilating forceps partially restored the breathing, but still the artificial obstruction was paramount. After animation had been well restored the tonsils were removed, followed by marked improvement in the breathing. The child remained in the ward of the hospital during the day. A Canadian medical man who happened to witness this case observed that a precisely similar case under gas occurred in his practice the previous week. If such cases teach any lesson, it is not the necessity for strychnine injection, which would have made matters worse in this case by stimulating the respiratory centres without enabling any air to reach the lung. It is that in cases of obstructed pharynx or larynx, especially in infants, the air passages should be cleared, and an artificial passage effected either by means of a bronchial catheter or a tracheotomy tube. Above all such cases teach the necessity for absolute unwavering attention to every case, and the necessity for prompt interference.

Some After Effects.

Ethyl chloride fell into disfavour owing to deaths having occurred suddenly and unexpectedly in several instances during its administration. The imperfect and crude methods of its administration and the use of impure ethyl chloride or ethyl chloride in combinations with other drugs were probably responsible for the catastrophes. The methods have now been greatly improved, the drug is well understood, and pure ethyl chloride can now be obtained from firms manufacturing it in this country—Messrs. Duncan, Flockhart and Company, of Edinburgh and London, and Hedley and Company, of Leytonstone. The after-administration procedure adopted at the Central London Throat Hospital is, perhaps, as simple as any. A room is set apart for patients recovering. As a rule the patient is placed in a chair or on a couch, recovers in two or three minutes, or even less, is sponged by the nurses in attendance and within a few minutes returns to his friends. Some take longer. If there is any difficulty the nurses raise the patient's legs, sponge the face with ice-water, and apply friction to the nose and mouth with a towel. In private practice this toilet should be done nicely and the recovery made agreeable and refreshing by spraying the face with rosewater or other pleasant fluid. In operations on the throat coughing cleanses the passages. An adult patient may have cold water to rinse the throat and mouth, but not children, as they are apt to swallow the fluid, and sickness and vomiting may be caused. In over six hundred administrations I have only seen one patient vomit in the operating-chair. That was a child whose mother had been warned not to give him any breakfast, and who, therefore, supplied him with two large oranges instead.

The recovery simulates the state of the patient at the onset of the administration: a screaming child will renew screaming on recovery; a struggling woman will struggle; a quiet, easy-going case will recover with equal ease. Thin, wiry women make the best recoveries, stout and lethargic persons take a little longer time. The patients are kept about fifteen minutes before returning to their friends, or a little longer in case of bleeding in nasal operations. Alcoholic patients are usually excitable and sometimes violent during recovery. They should be restrained as gently as possible, and kept in the chair until the recovery is complete.

During the administration unpleasant sensations are rare, but it should be noted that some patients have complained that the ethyl chloride causes erotic dreams and sensations.

My experience is similar to that of Dr. MacCardie. "For myself," he says, "I have not yet had a dangerous case in nearly fifteen hundred administrations." After-sickness is due to haste in administration and ill-timed endeavours to hasten the recovery of the patient or to the swallowing of blood. The one motto that should serve to guide practitioners in using this anæsthetic is to "use all gently." Be gentle with the gag, gentle with the application of the face-piece, gentle and watchful with the ethyl chloride, but, above all, be gentle with the recovering patient. There should be no undue haste in moving the patient from one chair to another or from the operating-chair to a couch. This increases vomiting and causes unnecessary struggling. Rest in the chair is the preventive of vomiting. Any vomiting that requires the injection of morphine or anything approaching the use of drugs of that nature is a pure chimera as far as ethyl chloride is concerned. The cause should be sought for elsewhere.

This advice does not imply that there is room in the administration of ethyl chloride for any remissness of observation or for want of promptitude in action should any untoward and unexpected occurrence arise, as it is apt to do with any anæsthetic.

It is maintained that all the deaths attributed to ethyl-chloride anæsthesia have occurred during the experimental stage of this anæsthetic. Incapable administrators are apt to ascribe dangers to the drug which should really be attributable in large part to themselves. Every experienced anæsthetist who has used ethyl chloride is unanimous as to its value and safety in suitable and experienced hands; but they are just as unanimous in condemning the grave risks and serious dangers attaching to the use of this anæsthetic agent by the unskilful or the reckless.

### Fatalities.

Statistics of the fatalities are difficult to collect and of very doubtful authenticity. Of seven cases reported by one collector, three deaths were due to the anæsthetic, although "not one of these three patients could be considered a favourable subject, and in two the administration of any anæsthetic was positively contraindicated." Of the remaining four cases death certainly was not due to the anæsthetic in one, and in another the disease was responsible. In the Edinburgh and Plymouth cases particulars have not been obtained. The accidents which are liable to occur are dangerous collapse, and symptoms of asphyxia. One would hesitate to give ethyl chloride in cases where dyspnœa was urgent, or mechanical obstruction even suspected. If patients are fat, lethargic and pronounced alcoholics, they are apt to suffer much from a slight deprivation of oxygen.

### A Longer Ethyl Chloride Anæsthesia.

One of the disadvantages of ethyl chloride is that the patient is liable to recover quite suddenly and without warning within a minute of the removal of the face-piece. This is rather disconcerting to an operator, and attempts have been made to devise some means of keeping up the supply of ethyl chloride vapour after the face-piece has been removed. This has been achieved to some extent by Dr. G. A. H. Barton, who has demonstrated a method for the prolonged administration of ethyl chloride, lasting, say, from five to ten, or perhaps even fifteen minutes. This will greatly enhance the value and the usefulness of ethyl chloride. The plan, however, involves considerable expense and a heavy strain on the administrator.

### Prolonged Anæsthesia.

While ethyl chloride is not an admirable anæsthetic for a very prolonged narcosis it is quite possible to keep up the anæsthesia for a considerable time. Whiteford has kept patients under ethyl chloride for 35 minutes, and Montgomery and Bland report a case of 54 minutes' anæsthesia. It appears, however, that when continued for more than 15 minutes it is apt to set up painful reaching and vomiting.

### The Ethyl Chloride Sequence.

Ethyl chloride is particularly suitable for beginning ether narcosis in alcoholic cases and in fat, double-chinned individuals, who are apt to be unnecessarily disturbed by ether or by "gas and ether." The ethyl chloride-chloroform sequence has been tried frequently and is recommended by some, but at the Central London Hospital the anæsthetists have discarded it. There is no reason, however, if the operator requires a long anæsthesia after beginning with ethyl chloride why the chloroform should not be given in the ordinary way with Junkers' apparatus, taking care at first not to push the chloroform beyond the lightest anæsthesia. Some anæsthetists administer ethyl chloride before chloroform in cases of timid and terrorised women and children, in whom it serves to cut short the dangerous stage of excitement.

Concluding Points.

While there is nothing particularly alarming in the administration of this new anæsthetic in educated hands it cannot be too deeply impressed upon practitioners that it is not a "glorified nitrous oxide which one can carry about in one's waistcoat pocket" and spray about indiscriminately without any adequate experience and skill in its administration. The careless and unskilled administrator will suddenly get a smart lesson which may make him drop the use of an agent whose effects are swifter both for good and evil than those he has been accustomed to, and lead him hastily, in the midst of his disappointment, to condemn the use of an agent otherwise capable of immense service. The practitioner should make it a point of duty to acquire the necessary instruction without which his experience may cost him dear.

## Figures and Tables

**Figure f1:**
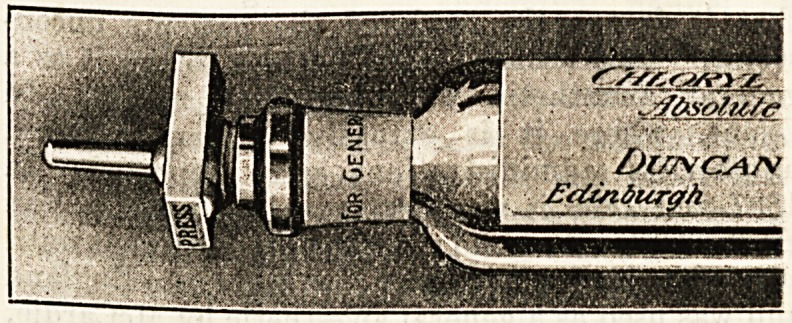


**Figure f2:**
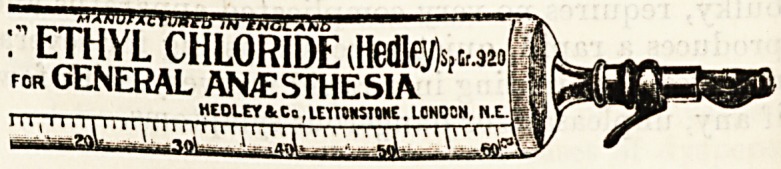


**Figure f3:**